# Influence of Pregnancy on the Occurrence of Lumbar Spine Pain in Polish Women: A Retrospective Study

**DOI:** 10.3390/jpm12030357

**Published:** 2022-02-26

**Authors:** Aleksandra Bryndal, Sebastian Glowinski, Marian Majchrzycki

**Affiliations:** 1 Department of Physiotherapy, Institute of Health Sciences, Pomeranian University, 76-200 Slupsk, Poland; sebastian.glowinski@tu.koszalin.pl; 2 Division of Mechatronics and Automatics, Faculty of Mechanical Engineering, Koszalin University of Technology, 75-453 Koszalin, Poland; 3 Division of Perinatology and Women’s Diseases, Poznan University of Medical Sciences, 61-701 Poznan, Poland; marian.majchrzycki@gmail.com

**Keywords:** pregnancy, low back pain, Roland Morris Disability Questionnaire, Oswestry Disability Index

## Abstract

(1) Background: Low Back Pain is a major health concern. Pregnancy-related lower back pain is a common complaint among women. The aim of this study was to determine the influence of pregnancy history on the occurrence and profile of pain in the lower spine. (2) Methods: The diagnosis of Lower Back Pain during pregnancy was based on the authors’ questionnaire, Visual Analogue Scale (VAS), Oswestry Disability Index and Roland Morris Disability Questionnaire. The research group consisted of 1112 women who were students or came for various reasons to the Gynecology and Obstetrics Clinical Hospital of Poznan University of Medical Sciences and completed the questionnaires. Patients were divided into two groups. The first group consisted of women who had never been pregnant (never-pregnant, *n* = 872 (78.4%), and the second group consisted of women who had been pregnant at least once in their lives (ever-pregnant, *n* = 240 (21.6%)). (3) Results: In the never-pregnant and the ever-pregnant women, respectively, the intensity of pain was 4.6 ± 1.6 and 5.0 ± 2.0 on the VAS scale, the degree of disability on the Oswestry Disability Index Questionnaire was 5.0 ± 3.9 and 5.5 ± 4.4, while the impact of pain on functioning on the Roland Morris Disability Questionnaire was 3.9 ± 3.1 and 3.9 ± 3.3. There were no significant differences (Mann-Whitney U test) between the studied groups in the parameters tested. (4) Conclusions: Pregnancy is a risk factor for back pain during pregnancy, but one year or more after pregnancy the occurrence of back pain is similar to that in women who have never been pregnant.

## 1. Introduction

Low back pain (LBP) is defined as pain in the lumbosacral part of the spine lasting at least one day with or without radiation to one or both lower limbs [1,2]. LBP is the leading cause of disability in adults of all ages. According to the World Health Organization (WHO), musculoskeletal diseases are the most common disabling conditions [3]. Approximately 75–80% of the world’s population will experience at least one episode of acute LBP in their lifetime. Most patients who develop acute LBP improve within approximately a month. However, many patients experience persistent symptoms of low-grade pain or recurrent episodes of LBP within one year of their last pain [4].

According to GBD 2017 (the Global Burden of Disease, Injuries and Risk Factors Study) worldwide the incidence of LBP according to age standardization was 7.50% [5]. The incidence was higher in women (8.01%) than in men (6.94%). The incidence of LBP increased with age, peaking at 80–89 years of age and then slightly decreasing. This pattern was observed in both women and men. LBP was the main reason for the number of years lived with disability (YLDs) among all conditions studied in GBD 2017. The number of years lived with disability was higher in women than in men [6].

There is now a growing interest in the influence of gender on pain experience. Women suffer from many chronic musculoskeletal aches and pain with an overwhelmingly greater frequency than men. Historically, these differences have been largely attributed to psychological factors. It has now been shown that biological factors, particularly hormonal mechanisms, play a major role in these differences. Pain sensitivity has been shown to vary throughout the menstrual cycle [7].

In addition to hormonal differences between sexes, pregnancy and childbirth can affect the musculoskeletal and neurological systems in various complex ways that can cause painful conditions. In particular, back and pelvic pain are common both during and after pregnancy [8,9]. Retrospective and prospective studies have estimated that 50–60% of pregnant women experience new back pain during pregnancy. In non-pregnant women aged 35 years, the rate is only 15% [10]. Therefore, pregnancy can be considered as an important risk factor for LBP in women of childbearing age. Many studies have suggested that lower back pain persists during the postpartum period [11,12]. Women with LBP during pregnancy have severe symptoms that interfere with work, sleep, and daily activities. In a retrospective cross-sectional study in 10–15% of women, it was shown that those who reported the onset of back pain during pregnancy still suffered from it [13]. It was also observed that in the 6th month after delivery, LBP occurs in 5–43%, and in the period of 3 years after childbirth, persistent pain did not subside in 20% of the women. It was strongly correlated with pain that started early in pregnancy and inability to reduce body weight to normal antenatal levels. The precise mechanisms by which musculoskeletal problems in the perinatal period contribute to chronic or future pain, including LBP, are poorly understood [12,14].

A high female body mass index is a risk factor for high-intensity LBP and LBP-related disability. Theories of the relationship between these include mechanical stresses on the lumbar spine due to weight factors, as well as the possible contribution of proinflammatory cytokines to adipose tissue. However, while some studies have reported an association between obesity and back pain, others have not [15,16]. Recently, an association between LBP and obesity has been observed in women, but not in men. The study of waist circumference as an indicator of obesity was more strongly associated with LBP in women than the body mass index. The reason why obesity is associated with back pain in women remains unknown. Indirect causes may be hormonal factors that play a role in obesity and also in pain modulation [17]. Understanding the factors that predispose women to LBP can have a large impact on public health. This can provide information on prevention strategies for a gender-specific society.

Earlier studies by our group examined the factors predisposing pregnant women to LBP based on questionnaire completion, and it was observed that pregnancy was a risk factor [18].

The aim of the present study was to determine the influence of pregnancy history on the occurrence and profile of pain in the lower spine and to analyze the impact of past pregnancies on the occurrence of pain in the lower spine and on the functioning and degree of disability.

## 2. Materials and Methods

The required sample size for research was determined using data from previous re-search (mean and standard deviation) [9]. We found that for 0.8 power and *p* < 0.05 the required number of people in each group should be 190.

**Figure 1:**
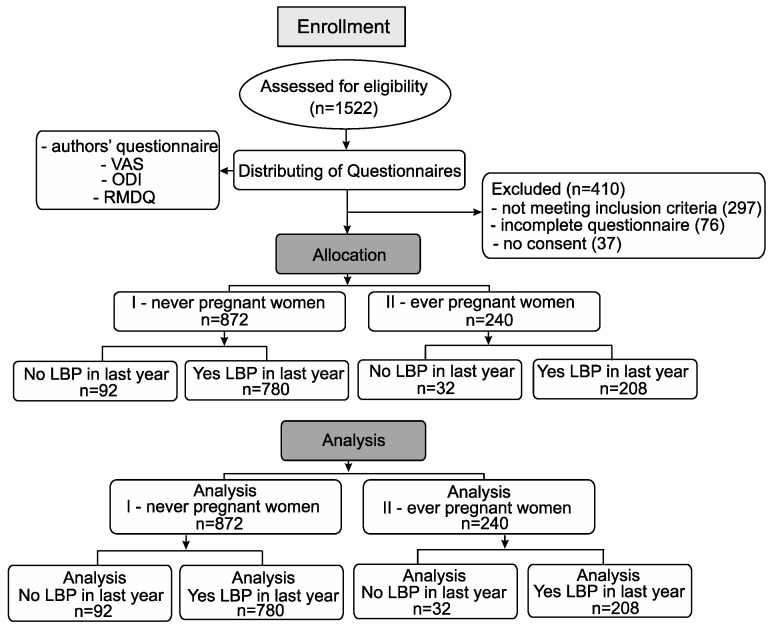
Flowchart of the experimental procedure.

The research group consisted of 1112 women who came for various reasons to the Gynecology and Obstetrics Clinical Hospital of Poznan University of Medical Sciences and students of the Poznan University of Medical Sciences (Poland). They completed questionnaires consisting of authors’ questionnaire, Visual Analogue Scale (VAS), the Oswestry Disability Index (ODI) and Roland Morris Disability Questionnaire (RMDQ). The questionnaire was conducted with 1522 women. A total of 410 pieces of data were excluded from the study due to failure to meet the inclusion criteria (*n* = 297), incomplete questionnaires (*n* = 76), and refusal to participate in the study (*n* = 37) (Figure 1). Data were collected using questionnaires designed to keep the personal information confidential. The questionnaires were distributed to hospital patients by midwives in paper form. The students were given these paper-based questionnaires at the university. All the participants were carefully instructed on how to complete the task. This study was conducted between April 2019 and March 2020.

The inclusion criteria for the studies were age in the range of 18–50 years, female gender, and no exclusion criteria, as follows:

- history of surgery of the spine in the lumbar region,
- the presence of cancer,
- spine deformities (e.g., scoliosis),
- osteoporosis,
- multiple sclerosis,
- fractures or any abnormalities in the pelvic area,
- inflammations,
- pregnancy while completing the questionnaire,
- less than a year passed since last pregnancy,
- refusal to participate in research.

All participants received written information about the purpose of the study and the possibility of discontinuation at any time. This study was approved by the Bioethics Committee of the Poznan University of Medical Sciences (No. 372/12).

The patients were divided into two groups. The first group consisted of women who had never been pregnant (never-pregnant) *n* = 872 (78.4%), and the second group consisted of women who had been pregnant at least once in their lives (ever-pregnant) *n* = 240 (21.6%). Both groups of women (never-pregnant and ever-pregnant) were divided into two more subgroups: women with LBP and women without LBP (Figure 1).

The authors’ questionnaire included questions regarding age, height, weight and number of pregnancies (if applicable). The questionnaire also included questions on the presence of LBP. Women with LBP were asked about their frequency of occurrence and whether LBP occurred before or during pregnancy (in the ever-pregnant group). The nature of LBP and the activities that trigger it were investigated.

The diagnosis of LBP is usually based on symptoms, owing to the few existing di-agnostic tests and scales. In the group of women with low back pain, the pain intensity was assessed using Visual Analogue Scale (VAS). The scale was in the form of a 10 cm ruler, on which the woman indicated with her finger or a slider the intensity of pain from 0—no pain at all to 10—the strongest pain imaginable [19,20]. Low back pain was scored independently while the patient was engaged in three different postural situations: motion, standing, and sitting.

In the group of women with low back pain (never-pregnant with LBP *n* = 780 and ever-pregnant with LBP *n* = 208), the degree of disability caused by back pain in the lumbar region was also determined. For this purpose, the Oswestry Disability Index (ODI) questionnaire was used. This is now a standard questionnaire to assess the impact of back pain on patients’ daily activities. The ODI questionnaire is reliable, acceptable, and sensitive to changes in patient assessment [21,22]. Each woman completed the questionnaire on her own, answering 10 questions concerning: pain intensity and variability in time, lifting objects, sitting, sleeping, traveling, caring, walking, standing, socializing, and changing the intensity of pain. In each section of the questionnaire, each woman chose only one answer that best described the current validity, scored from 0, no limits to 5, maximum limits. The maximum number of points was 50. The degree of disability was determined as a percentage (the higher the score, the greater the disability). The patients were divided into five groups according to the degree of disability expressed as a percentage. Minimal disability—0–20%, moderate disability—21–40%, severe disability—41–60%, crippling disability—61–80%, and bed-bound patient—81–100%. Women can cope with most of their daily activities with minimal disability. Usually, no treatment is indicated, apart from advice on lifting sitting and exercise. In cases of moderate disability, the patient experiences more pain and difficulty in sitting, lifting, and standing. Moreover, in this case, travel and social life were more difficult, whereas personal care, sexual activity and sleep were not grossly affected. Patients can usually be managed by conservative means. Patients with severe disability require a detailed investigation and their activities of daily living are affected. In crippling disabilities, back pain impinges on all aspects of a woman’s life. In the last stage of disability, patients are either bed-bound or exaggerating their symptoms.

In the group of women with pain, the influence of lower back pain on functioning was analyzed. This was done using the Roland Morris Disability Questionnaire (RMDQ). This consists of 24 sentences concerning everyday activities to which the patient answers “yes” or “no”. Each woman completed the questionnaire on her own (5–10 min to complete) by choosing the sentences that best described her functioning on the day of completing the questionnaire (a maximum of 24 tasks could be selected). The results ranged from 0, no disability, to 24, high disability. The patients were divided into four groups depending on the number of points obtained: no disability—0–3 points, low level of disability—4–10 points, medium—11–17 points, high—18–24 points [19,21,23,24,25]. All questionnaires used in this study had already been translated into Polish, and their reliability and validity had been tested and approved [22,25].

All women participating in the study were asked to complete the questionnaires accurately and reliably.

Statistical calculations were performed using StatSoft statistical package. Inc. (New York, NY, USA) (2020) STATISTICA version 13.3 [26].

All analyzed data for continuous variables were characterized by the arithmetical mean, standard deviation, median, minimum and maximum values (range), and a 95% confidence interval (CI). At the outset, we examined whether there were outliers in all variables using the Grubbs test. This made it possible to verify the accuracy of the data. To determine whether the continuous variable came from a normally distributed population, the following tests were used: W Shapiro-Wilk, Lilliefors, Kołomogorow-Smirnow, and Jarque-Bera.

The Brown–Forsythe (Leven) test was used to test the hypothesis of equal variance. The significance of differences between the two groups (model of unrelated variables) was tested using tests of significance of differences: Student’s *t*-test (or Welch’s test in the case of lack of homogeneity of variance) or the Mann-Whitney U test (in the case of failure to meet the Student’s *t*-test applicability conditions).

To determine the relationship between strength and direction of the variables, a correlation analysis was performed to calculate Pearson’s correlation coefficients. Before starting the study of the interrelationships between variables, charts were drawn to illustrate the strength and direction of their relationships. This allowed us to determine whether the points deviated from the others. For all calculations, *p* = 0.05 was adopted as the level of significance.

## 3. Results

**Figure 2:**
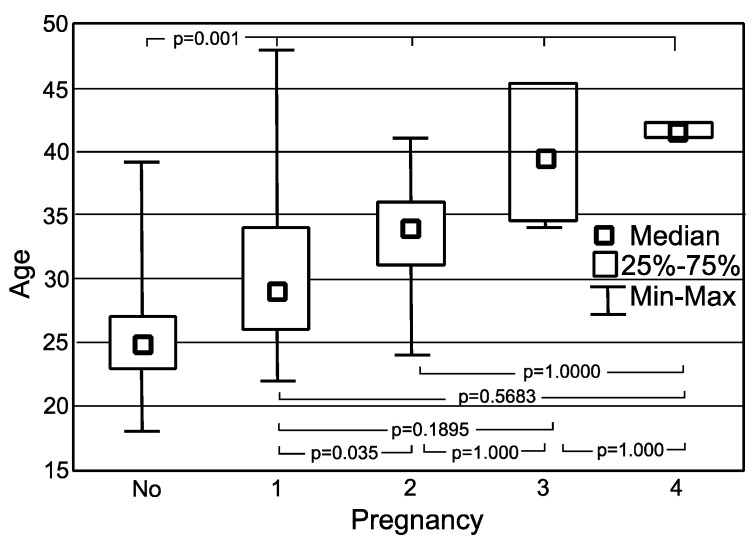
Age by number of pregnancies in the study group.

Table 1 presents the characteristics of the research group (*n* = 1112) along with their division into subgroups. The first group consisted of women who had never been pregnant, while the second group consisted of women who had been pregnant at least once in their life, and at the time of completing the questionnaire they were at least one year from their last pregnancy (Figure 1). The minimum age of participation in the study was 18 years, and the maximum 48 years, respectively (Figure 2).

The results of the Kruskal-Wallis test showed statistically significant difference between the ages of non-pregnant and ever-pregnant women (*p* = 0.0001 up to *p* = 0.0011, respectively). In the case of women who had been 1 time pregnant, the difference in age was not statistically significant when comparing 3 and 4 times pregnant (*p* = 0.1895 and *p* = 0.5683). The same situation applied for women who were 2 times pregnant (*p* = 1.0000). It can be concluded that women with a greater number of pregnancies are older than women with 0 and 1 pregnancies, which seems understandable (Figure 2).

**Figure 3:**
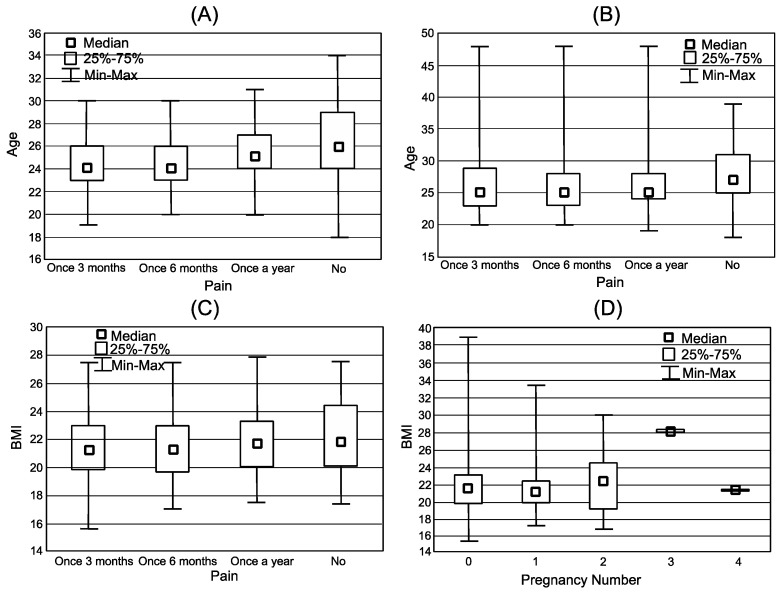
Box plot representation of the distribution of women (**A**) Age vs. pain frequency—never pregnant; (**B**) Age vs. pain—ever pregnant; (**C**) BMI vs. Pain—never pregnant; (**D**) BMI vs. Pain-ever pregnant.

There was a statistically significant difference between the groups (regarding pain) in terms of age, but no significant differences were found in height, weight, or BMI (Body Mass Index) (Table 1). There was no statistically significant difference in age between the never-pregnant and ever-pregnant groups in the number of pain episodes (Figure 3A,B).

In the group of ever-pregnant women, 60% had been pregnant once, 35% twice, and 5% three or more times. 70% of ever-pregnant women declared that they had experienced back pain during pregnancy, 55% had experienced this before pregnancy, 21% were pregnant for the first time, and 24% were unable to determine the onset of pain occurrence.

**Figure 4:**
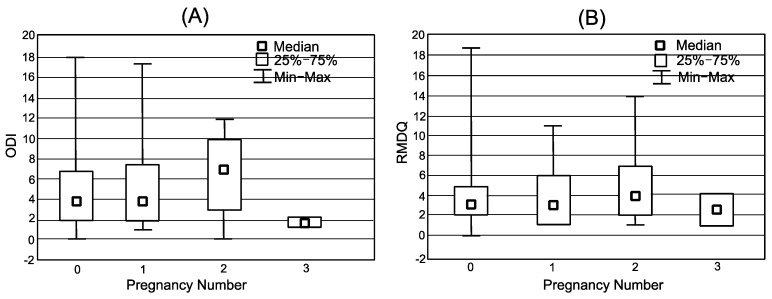
Relationship between number of pregnancies and pain intensity (**A**) ODI = the Oswestry Disability Index; (**B**) RMDQ = the Roland Morris Disability Questionnaire.

In group II (ever-pregnant), no significant correlation was observed between the intensity of pain as a result of LBP and age, the degree of disability (ODI), the number of pregnancies; or between the effect of pain on functioning (RMDQ), the number of pregnancies and BMI and the number of pregnancies (Figure 4).

Table 2 presents the percentage of pain in the entire study group, which was divided into two sub-groups. The percentage of responses to a given question was presented for the entire study group in group I and II. In both groups, women who had experienced low back pain in the past year were selected. Low back pain was also characterized in women who reported back pain within the last month of participating in the study. The Fisher, Chi-square and Pearson Chi-square tests showed no statistically significant differences between the groups.

The obtained data show that, in the entire group of women with LBP, the intensity of pain is 4.7 ± 1.7 on the VAS scale, the degree of disability (ODI) is 5.2 ± 4.0, and the impact of pain on functioning (RMDQ) is 3.9 ± 3.2. In the first group of women who were not pregnant and the second group of pregnant women, the intensity of pain was determined as 4.6 ± 1.6; 5.0 ± 2.0 on the VAS scale, the degree of disability (ODI) was 5.0 ± 3.9 and 5.5 ± 4.4 respectively, while the impact of pain on functioning (RMDQ) was 3.9 ± 3.1 and 3.9 ± 3.3. There were no significant differences (Mann-Whitney U test) between the studied groups in the parameters tested.

## 4. Discussion

The aim of this study was to determine the influence of pregnancy history on the occurrence and profile of pain in the lower back, considering factors such as number of pregnancies, pain characteristics (time of day, activities causing pain), age, weight, and BMI.

Many scientific reports have examined the occurrence and treatment of back pain during pregnancy [7,9,18,23,24,27,28,29,30], but the authors did not find any reports comparing the occurrence of LBP in women who were pregnant with women who were not pregnant.

In studies by Ostgaard et al. 1991 [10] in a group of women aged 35 years who were pregnant, 50–60% of them experienced pain, while in the same age group women who were not pregnant experienced back pain in only 15% of cases. In our study, the incidence of LBP by self-report was similar in both groups. In the group of pregnant women aged 31.8 years on average, pain was reported by 87%, whereas in the group of women who were not pregnant aged 25.3 years on average, pain was re-ported by 89%. In this study, the women who were never pregnant were younger than those who were pregnant. This age difference may explain the similar percentage of LBP occurrence one year after completing the questionnaire in both groups. Kristiansson et al. [31] reported no association between age and LBP, whereas Ostgaard et al. [10] and Bryndal et al. [9] found that young mothers had LBP more frequently than older mothers. This may indicate that younger women are more likely to experience LBP, which coincides with the high prevalence of LBP in nonpregnant women. Moreover, pregnancy predisposes women to later LBP development, which may lead to a higher LBP score in women who have been pregnant in the past. Research is needed on groups of women with a history of pregnancy and those without pregnancy in a similar age group. The association between age and LBP remains controversial, both among women who were screened during pregnancy and those with and without a history of pregnancy. In another study by Mens et al. [32], there was no relationship between BMI and LBP, whereas in a multicenter study BMI ≥ 30 kg/m^2^ was associated with LBP [33]. As indicated by Koyanagi et al. [15] and Shiri et al. [16], female weight influences the incidence of LBP. However, when analyzing the influence of BMI on the occurrence of pain in both groups (ever-pregnant and never-pregnant), no significant relationship was observed. In this study, we found no relationship among age, BMI, and LBP. The different results regarding the relationship between age and BMI and LBP may be explained by the different sample sizes in previous studies as well as the diversity of the genetic factors involved in women. Genetic factors have also been described as predisposing factors for disc degeneration [34], which is one of the causes of LBP [35].

In the entire study group of women, the degree of disability (ODI) was on average 5.2. The impact of pain on functioning (RMDQ) was estimated at 3.9 on average. These results did not differ significantly between both groups (never pregnant and ever pregnant). Results demonstrate the minimal influence of LBP on functioning. As noted by the presence of LBP, sitting at 21%, lifting—16%, standing at 15%, bending (14%, walking—13%, exercising—11%, and lying/sleeping—10%) are difficult, which may affect the ODI and RMDQ results. Difficulties in performing these activities, particularly when they are permanent, should not be ignored.

There was no effect of the number of pregnancies on the ODI and RMDQ results. As noted earlier, the ODI and RMDQ results were not significantly different between the two groups of women who were pregnant and who were not pregnant.

This study has some limitations. We did not collect data on the exact time since the last pregnancy or LBP duration. Moreover, the cross-sectional design did not allow the establishment of causality. However, the positive aspects of the study were the large sample and the results themselves, which showed the importance of stratification by sex and past pregnancies.

## 5. Conclusions

Pregnancy is a risk factor for back pain during pregnancy, but at least one year after pregnancy the occurrence of back pain is similar to that in women who have never been pregnant. In our analysis, no significant differences were found in LBP incidence in women who had never been pregnant compared to women who had been pregnant at least once, which was nearly 89% for back pain at least once a year. The degree of disability, impact of pain on function, nature of pain, and the time it occurred were similar in both study groups. This study can be used as a benchmark for other epidemiological studies and to increase the knowledge of national estimates of LBP prevalence and risk factors by gender and pregnancy. Prospective studies with valid and reliable tools are needed to estimate LBP risk factors by gender and pregnancy history.

## Figures and Tables

**Table 1:** Basic characteristics of the studied variables divided into groups.

	All Group *n* = 1112	I Never Pregnant Woman *n* = 872	II Ever Pregnant Woman *n* = 240	*p*-Value
Age (years)	26.7 (4.8)	25.3 (3.3)	31.8 (5.8)	0.0000 ^1^
	18.0–48.0	18.0–39.0	22.0–48.0	
	25.0	25.0	31.0	
	[26.4; 26.9]	[25.0; 25.5]	[31.1; 32.5]	
Height (cm)	167.6 (6.1)	167.6 (6.0)	167.8 (6.5)	0.9781 ^1^
	152.0–185.0	152.0–185.0	157.0–185.0	
	167.0	167.0	168.0	
	[167.3; 168.0]	[167.2; 168.0]	[167.0; 168.6]	
Weight (kg)	61.6 (10.2)	61.3 (10.1)	62.5 (10.4)	0.0912 ^1^
	40.0–119.0	40.0–119.0	45.0–100.0	
	60.0	60.0	60.0	
	[61.0; 62.2]	[60.6; 62.0]	[61.2; 63.8]	
BMI (kg/m^2^)	21.9 (3.0)	21.8 (2.9)	22.2 (3.2)	0.2558 ^1^
	15.6–39.0	15.6–39.0	17.0–33.4	
	21.5	21.5	21.4	
	[21.7; 22.0]	[21.6; 22.0]	[21.7; 22.6]	

^1^ U Mann-Whitney test.

**Table 2:** Characteristics of the participants in terms of the occurrence of pain.

			All Group *n* = 1112	Never-Pregnant *n* = 872	Ever-Pregnant *n* = 240
	Occurrence of low back pain in last year (%)	No	124 (11%)	92 (11%)	32 (13%)
		Yes	988 (89%)	780 (89%)	208 (87%)
	Pain frequency (%)	No	124 (11%)	92 (11%)	32 (13%)
		Yes—once a year	272 (24%)	219 (25%)	53 (22%)
		Yes—once per 6 months	274 (25%)	210 (24%)	64 (27%)
		Yes—once per 3 months	442 (40%)	351 (40%)	91 (38%)
Occurrence of low back pain	The pain nature (%)	constant	73 (18%)	44 (15%)	29(23%)
		temporary	191 (46%)	137 (47%)	54 (44%)
		local	112 (27%)	79 (27%)	33 (27%)
		radiating	36 (9%)	28 (10%)	8 (6%)
		other	4 (1%)	4 (1%)	0 (0%)
	Time of occurrence of pain symptoms (%)	in the morning	83 (15%)	44 (12%)	39 (23%)
		during the day	259 (48%)	184 (48%)	75 (44%)
		in the night	66 (12%)	66 (12%)	30 (18%)
		after exercise	137 (25%)	137 (25%)	26 (15%)
	Activities essential in everyday life, the performance of which is difficult due to pain (%)	bending down	112 (14%)	68 (12%)	44 (15%)
		lying down sleeping	80 (10%)	60 (11%)	80 (28%)
		seating	163 (21%)	133 (24%)	30 (10%)
		standing	118 (21%)	91 (16%)	27 (9%)
		walking	105 (13%)	70 (12%)	35 (12%)
		physical activity	87 (11%)	57 (10%)	30 (10%)
		lifting	130 (16%)	84 (15%)	46 (16%)

## Data Availability

The data presented in this study are available on request from the corresponding author.

## References

[1] Hoy D., Bain C., Williams G., March L., Brooks P., Blyth F., Woolf A., Vos T., Buchbinder R. (2012). A systematic review of the global prevalence of low back pain. Arthritis Rheum..

[2] Glowinski S., Krzyzynski T. (2013). Modelling of the ejection process in a symmetrical flight. J. Theor. Appl. Mech..

[3] World Health Organization (2008). The Global Burden of Disease: 2004 Update.

[4] Hartvigsen J., Hancock M.J., Kongsted A., Louw Q., Ferreira M.L., Genevay S., Woolf A. (2018). What low back pain is and why we need to pay attention. Lancet.

[5] Institute for Health Metrics and Evaluation. http://www.healthdata.org/gbd/data.

[6] Wu A., March L., Zheng X., Huang J., Wang X., Zhao J., Blyth F.M., Smith E., Buchbinder R., Hoy D. (2020). Global low back pain prevalence and years lived with disability from 1990 to 2017: Estimates from the Global Burden of Disease Study. Ann. Transl. Med..

[7] Chen L., Ferreira M.L., Beckenkamp P.R., Caputo E.L., Feng S., Ferreira P.H. (2021). Comparative Efficacy and Safety of Conservative Care for Pregnancy-Related Low Back Pain: A Systematic Review and Network Meta-analysis. Phys. Ther..

[8] Riley J.L., Robinson M.E., Wise E.A., Price D. (1999). A meta-analytic review of pain perception across the menstrual cycle. Pain.

[9] Bryndal A., Majchrzycki M., Grochulska A., Glowinski S., Seremak-Mrozikiewicz A. (2020). Risk factors associated with low back pain among a group of 1510 pregnant women. J. Pers. Med..

[10] Ostgaard H.C., Andersson G.B.J., Karlsson K. (1991). Prevalence of back pain in pregnancy. Spine.

[11] Sakamoto A., Nakagawa H., Nakagawa H., Gamada K. (2018). Effect of Exercise with a Pelvic Realignment Device on Low-Back and Pelvic Girdle Pain After Childbirth: A Randomized Control Study. J. Rehabil. Med..

[12] Komatsu R., Ando K., Flood P.D. (2020). Factors associated with persistent pain after childbirth: A narrative review. Br. J. Anaesth..

[13] Matsuda N., Kitagaki K., Perrein E., Tsuboi Y., Ebina A., Kondo Y., Ono R. (2020). Association between excessive weight gain during pregnancy and persistent low back and pelvic pain after delivery. Spine.

[14] Noren L., Ostgaard S., Johansson G. (2002). Lumbar back and posterior pelvic pain during pregnancy: A 3-year follow-up. Eur. Spine J..

[15] Koyanagi A., Stickley A., Garin N., Miret M., Ayuso-Mateos J.L., Leonardi M., Haro J.M. (2015). The association between obesity and back pain in nine countries: A cross-sectional study. BMC Public Health.

[16] Shiri R., Karppinen J., Leino-Arjas P., Solovieva S., Viikari-Juntura E. (2009). The Association between Obesity and Low Back Pain: A Meta-Analysis. Am. J. Epidemiol..

[17] Bailey A. (2009). Risk factors for low back pain in women. Menopause.

[18] Carlson H.L., Carlson N.L., Pasternak B.A., Balderston K.D. (2003). Understanding and managing the back pain of pregnancy. Curr. Womens Health Rep..

[19] Kent P., Lauridsen H.H. (2011). Managing Missing Scores on the Roland Morris Disability Questionnaire. Spine.

[20] Carlsson A.M. (1983). Assessment of chronic pain. I. Aspects of the reliability and validity of the visual analogue scale. Pain.

[21] Roland M.O., Morris R.W. (1983). A study of the natural history of back pain. Part 1: Development of a reliable and sensitive measure of disability in low back pain. Spine J..

[22] Miekisiak G., Kollataj M., Dobrogowski J., Kloc W., Libionka W., Banach M., Latka D., Sobolewski T., Sulewski A., Nowakowski A. (2013). Validation and Cross-Cultural Adaptation of the Polish Version of the Oswestry Disability Index. Spine.

[23] Quintero Rodriguez C., Troynikov O. (2019). The Effect of Maternity Support Garments on Alleviation of Pains and Discomforts during Pregnancy: A Systematic Review. J. Pregnancy.

[24] Sehmbi H., D’Souza R., Bhatia A. (2017). Low Back Pain in Pregnancy: Investigations, Management, and Role of Neuraxial Analgesia and Anaesthesia: A Systematic Review. Gynecol. Obstet. Investig..

[25] Opara J., Szary S., Kucharz E. (2006). Polish Cultural Adaptation of the Roland-Morris Questionnaire for Evaluation of Quality of Life in Patients with Low Back Pain. Spine.

[26] Statistica. https://www.statsoft.pl.

[27] de Campos T.F., Maher C.G., Fuller J.T., Steffens D., Attwell S., Hancock M.J. (2021). Prevention strategies to reduce future impact of low back pain: A systematic review and meta-analysis. Br. J. Sports Med..

[28] Ferreira C., Alburquerque-Sendín F. (2012). Effectiveness of physical therapy for pregnancy-related low back and/or pelvic pain after delivery: A systematic review. Physiother. Theory Pract..

[29] Wiezer M., Hage-Fransen M.A.H., Otto A., Wieffer-Platvoet M.S., Slotman M.H., Nijhuis-Van der Sanden M.W.G., Pool-Goudzwaard A.L. (2020). Risk factors for pelvic girdle pain postpartum and pregnancy related low back pain postpartum; A systematic review and meta-analysis. Musculoskelet. Sci. Pract..

[30] Pennick V., Liddle S.D. (2013). Interventions for Preventing and Treating Pelvic and Back Pain in Pregnancy. Cochrane Database Syst. Rev..

[31] Kristiansson P., Svärdsudd K., von Schoultz B. (1996). Serum relaxin, symphyseal pain, and back pain during pregnancy. Am. J. Obstet. Gynecol..

[32] Mens J.M., Vleeming A., Stoeckart R., Stam H.J., Snijders C.J. (1996). Understanding peripartum pelvic pain. Implications of a patient survey. Spine.

[33] Mogren I.M., Pohjanen A.I. (2005). Low back pain and pelvic pain during pregnancy: Prevalence and risk factors. Spine.

[34] Toktaş Z.O., Ekşi M.Ş., Yılmaz B., Demir M.K., Özgen S., Kılıç T., Konya D. (2015). Association of collagen I, IX and vitamin D receptor gene polymorphisms with radiological severity of intervertebral disc degeneration in Southern European Ancestor. Eur. Spine J..

[35] Kawaguchi Y., Kanamori M., Ishihara H., Ohmori K., Matsui H., Kimura T. (2002). The association of lumbar disc disease with vitamin-D receptor gene polymorphism. J. Bone Joint Surg. Am..

